# Epidemiology of Traumatic Brain Injury in Urmia, Iran

**DOI:** 10.5812/ircmj.2090

**Published:** 2013-02-05

**Authors:** Nader Aghakhani, Mehdi Azami, Madine Jasemi, Mohammad Khoshsima, Samereh Eghtedar, Narghes Rahbar

**Affiliations:** 1 Department of Nursing, Urmia University of Medical Sciences, Urmia, IR Iran; 2 Professor Alborzi Clinical Microbiology Research Center, Shiraz University of Medical Sciences, Shiraz, IR Iran; 3 Health Policy Center, Shiraz University of Medical Sciences, Shiraz, IR Iran

**Keywords:** Epidemiology, Head injuries

**Dear Editor,**

Traumatic brain injury (TBI) is a major cause of morbidity and mortality after myocardial infarction in the world with many complications in somatic, psychosocial and disabilities(1). Each year in the United States, around 50,000 people die after a TBI. This accounts for 1/3 of all injury deaths. In addition, 90,000 people experience lifelong disability associated with a TBI(2). The overall number of TBI cases has declined over the years following legislation as well as public education about preventive measures such as the use of helmets, the enforcement of passenger and child restraints, speed limits and tough drink driving penalties. Coupled with improvements in emergency medical evacuation, neurosurgical and intensive care, these changes have meant that more patients are surviving injuries that would have previously been fatal(3). The most frequent cause associated with both fatal and non-fatal brain injury is transport-related. It involves motor vehicles, bicycles, motorcycles and pedestrian-automobile accidents and accounts for around 50% of all accidents(4).

The second most significant cause of TBI is falls, which accounts for 20% to 30% of all injuries, especially among the very young and the elderly. In many parts of the world, assault is fast becoming one of the leading causes of brain trauma, particularly in the lower socio-economic groups and war-torn countries. Other significant causes include the use of firearms (around 12%) and sports or recreational related activities(5). The risk of TBI is highest in the 15-24-year-old age group and decreases in the midlife years, but rises again after 70 years. A second peak occurs in the elderly due to falls(4).

We under took a population-based epidemiologic to study TBI in urban and rural areas in West Azarbaijan province in 2005-2006. In this retrospective study,we reviewed TBI-patients records in Motahhari hospital in Urmia during 2005 to 2006 years. The medical records based on the ICD items, TBI-related death based on the death certificate and demographic data were collected.

From the total of 1796 hospitalized patients for TBI, 721 patients (40.1%) have acute TBI that 1392 (77.5%) of them were males. The maleratio to female was 3 to 1. Most of patients (395=54.8%) lived in Urmia and remaining patients lived in Khoi (17.8%), Miandoab (13.3%) and Salmas (14.1%). The most common age group was 20-29 years (24.3%). The most frequent mechanism of trauma was motor-vehicle accident. 81.7% of injuries were mild in severity. Mean age of dead persons was 31.9 years old. Most of injuries were in Farvardin, because of trips were increasing this time. Most of the victims were motorcyclist (42%). Accidents rate in road was more than urban streets and most of them happened in spring and summer season, respectively. Most of the mortalities took place in the first minutes and in place of accident (66%).

This study showed that 40.1% of all patients were hospitalized for acute TBI. The findings of this study are similar to those reported in the Chicago (40%)(6)and Bronx County (34%)(7). Most of patients were males which is similar to results of Colorado reported(8). The most common age group was 20-29 years that this results are similar to other studies(2,3). In Taiwan also the highest incidence of TBI shifted to the 20–29 years of age that is similar to our study and it may reflect the transformation of Asian countries into a more Western-like environment(9).Our results showed that the main cause of accidents was vehicles and most of the victims were motorcyclist. Accidents rate in road was 57.2%. Literature review showed that traffic accidents are the major cause of injury (77%) in Iran that 48% of them were motorcyclist(10). Road accidents in Iran have more frequent than other country. The motorcycle has been the major way of transportation in Taiwan and it's the major cause of mortality in this area(9). In this study, the mortality rate was 0.5% and 81.7% of injuries were mild in severity. Review of literature show that Most of the accidents took place is during New Year holiday. In our study,most of accidents happened in Farvardin(March), it may reflect the increase of trips in the region at Noroozholiday.

The findings demonstrate that the lack of a system to road-user safety was the main cause of injury; therefore the focus of all activities should take place in this field. It seems that more attention is necessary for researchers and health-policy makers to published TBI related articles to increase public knowledge in Iran. It must be remembered that prevention of TBI is vital as there remains no cure for the moderate-to severe TBI.Asthe evidence for effectiveness and specific treatment is limited so it must be subjected to rigorous research.
